# Development of Ultra-High-Density Screening Tools for Microbial “Omics”

**DOI:** 10.1371/journal.pone.0085177

**Published:** 2014-01-21

**Authors:** Gordon J. Bean, Philipp A. Jaeger, Sondra Bahr, Trey Ideker

**Affiliations:** 1 Bioinformatics and Systems Biology Program, University of California San Diego, La Jolla, California, United States of America; 2 Departments of Medicine and Bioengineering, University of California San Diego, La Jolla, California, United States of America; 3 Banting and Best Department of Medical Research, Terrence Donnelly Centre for Cellular and Biomolecular Research, University of Toronto, Toronto, Canada; 4 Institute for Genomic Medicine, University of California San Diego, La Jolla, California, United States of America; Deutsches Krebsforschungszentrum, Germany

## Abstract

High-throughput genetic screens in model microbial organisms are a primary means of interrogating biological systems. In numerous cases, such screens have identified the genes that underlie a particular phenotype or a set of gene-gene, gene-environment or protein-protein interactions, which are then used to construct highly informative network maps for biological research. However, the potential test space of genes, proteins, or interactions is typically much larger than current screening systems can address. To push the limits of screening technology, we developed an ultra-high-density, 6144-colony arraying system and analysis toolbox. Using budding yeast as a benchmark, we find that these tools boost genetic screening throughput 4-fold and yield significant cost and time reductions at quality levels equal to or better than current methods. Thus, the new ultra-high-density screening tools enable researchers to significantly increase the size and scope of their genetic screens.

## Introduction

Large-scale genetic screening experiments (i.e. simultaneous analysis of many mutants, either pooled or arrayed) have enabled researchers to identify gene functions and functional relationships underlying many processes (for numerous examples see *Nature Reviews Genetics* series “The Art and Design of Genetic Screens”). In an increasing number of model organisms, such screens take advantage of available mutant libraries, including complete collections of gene knock-out strains, over-expression constructs, and the like. In the most typical mode, these screens identify genes that are required for, or modulate, a phenotype of interest. Very similar screens can be performed to identify gene and protein interactions using systems such as Synthetic genetic arrays (SGA) and Yeast two-hybrid (Y2H)[1]–[5].

In this context, single-cell organisms have proven extraordinarily useful due to their ease of genetic manipulation and straightforward growth conditions. Suitable species can be found in bacteria (e.g. *E. coli*), fungi (e.g. *S. cerevisiae, S. pombe*), and algae (*C. reinhartdii*), allowing researchers to assess the effects of a gene across large evolutionary timescales [6]–[9]. However, even the comparatively small genomes of these model species contain thousands of genes that can be screened in any number of growth conditions. Furthermore, screening for combinations of mutants, such as in genetic interaction screening or physical interaction mapping, requires hundreds of thousands to millions of possible strains.

Most current screening methods rely on growing microbial model organisms on a solid, nutrient-rich agar surface in a regular grid pattern to allow for reliable parallel quantification of a simple phenotype such as growth [6]–[8]. The maximum density of microbial colonies per surface unit, the duration of necessary growing time, and the sensitivity and robustness of the downstream image acquisition and analysis pipeline are all important factors that determine screening throughput, and present screening systems typically allow for up to 1536 colonies per agar plate [10].

Here, using *S. cerevisiae* as a benchmark, we substantially enhance screening throughput by enabling growth and analysis of 6144 mutant yeast colonies on a single agar plate. The significance of achieving this number is that the vast majority of microbial model organisms have gene counts very near but not exceeding this number, allowing for an entire, genome-wide screen to be performed on a single agar plate. We evaluate data quality and cost performance of this new, ultra-high-density colony-transfer system in comparison to current methods, and provide a free computational toolset for ultra-high-density image analysis.

## Materials and Methods

### 6144-density pad development

The new 6144-density pads were produced in collaboration between Singer Instrument Co. Ltd. (Roadwater/UK), KREO Technologies (Oakville, ON/Canada) and S.B. in the Boone laboratory (University Toronto, ON/Canada). Different pressure molds were cut and several trial pads were cast by varying plastic temperature, injection pressure etc. Pads were evaluated for flatness, stiffness, and pinhead quality using standard, SingerPlus plates as well as aluminum and polytetrafluoroethylene SingerPlusPlate^+^ prototypes. The pad with the overall best performance and tolerance was chosen for production and is now commercially available (Singer Instrument Co. Ltd.).

### Yeast deletion strains, agar plates, and media preparation

The yeast strains used in this report are based on the commercially available yeast knockout (YKO) strain collection (Thermo Fisher Scientific Inc., Waltham/MA) with kanamycin as a deletion marker. The collection was stored in glycerol stocks at −80°C in 96-well format until used. We produced higher-density plates by first pinning thawed glycerol stocks onto agar plates and then robotically combining 96 plates into increasingly higher densities. Media and agar plates were composed following established E-MAP protocols [6]–[8], [10]–[12] in standard Singer plate clones (IGENE Supplies, Shanghai/China). Microtiter-format agar plates were poured manually with 42 ml of liquid agar-medium in each plate, cooled on the bench top overnight, and were allowed to dry for 24 hrs at room temperature. It is critical for high-density pinning that the agar surface is dry before pinning starts.

### 1536- and 6144-density-format pinning

To achieve estimates of technical and biological variances, we pinned a minimum of 18 replicate plates of each format and imaged each plate at 0, 3, 6, 9, 12, 24, and 48 hours after pinning (the 96 and 384 plates were not imaged on the 3- and 9-hour time points). Additionally, we imaged every plate's source plate immediately before pinning. Overall, we acquired over 1200 high-resolution plate images. Unless otherwise specified, measurements and analyses using the 6144 format were done on the 12-hour images, while measurements and analyses using the 384- and 1536 formats were done using the 48-hour images.

All liquid-to-solid and solid-to-solid yeast transfers were conducted using a Singer RoToR robotic plate handler (Singer Instrument Co. Ltd). 96- to 1536-format pinnings were performed using the respective factory standard settings for source and target plates. 1536-to-6144 (1536x4) pinnings with a 1536 pad were performed with default factory settings at the source plate (with 0.15 mm offset) and custom settings for the target plate (pin pressure 64%, speed 10 mm/s, overshoot 1 mm, no offset). 6144-to-6144 pinnings with a 6144 pad were performed with custom settings at the source (pin pressure 50%, speed 10 mm/s, overshoot 0.6 mm, no offset) and the target plate (pin pressure 64%, speed 10 mm/s, overshoot 0.6 mm, no offset).

For clean 1536-to-6144, 6144-to-6144, and 6144-to-24576 transfers it is essential not to overgrow the source plates (max. 6–12 hrs incubation) as the pinheads are very small and overly large source colonies lead to cross-contamination and smear formation between neighboring colonies. For hyper-density plates, we first pinned 6144 source plates, incubated those for only 3 hrs at room temperature and the immediately pinned again to 24576.

### Digital image acquisition

All digital images were acquired with a commercially available SLR camera (18Mpixel Rebel T3i, Canon USA Inc., Melville/NY) with an 18–55 mm zoom lens. We used a white diffusor box with bilateral illumination and an overhead mount for the camera in a dark room. Images were taken in highest quality, 8-bit JPEG. Down-sampling experiments suggest that 10Mpixel cameras should be sufficient for 6144-format image acquisition (data not shown).

### Image analysis and data processing

Images were normalized, spatially corrected, and quantified using a set of custom algorithms (aka “The Colony Analyzer Toolkit”) written in Matlab (MathWorks Inc., Natick/MA). The complete software package is available online (https://github.com/brazilbean/Matlab-Colony-Analyzer-Toolkit). The workflow for measuring colony size from digital images is as follows: (1) the image is cropped to the plate, (2) the colony grid is overlaid on the plate image, (3) the size of each colony is measured, (4) colony sizes are plate normalized, and (5) colony sizes are spatially corrected to remove local, nutrient-based growth effects. Colony size quantification and analysis generally followed the same procedures used in other studies [6], [11]. However, the toolkit can be easily adapted to measure other features besides size, such as color or average pixel intensity. An additional version of the toolkit, written in Python, is under development and will be made available at https://github.com/brazilbean/bean-python-toolkit.

## Results

### Technical and computational improvements required for ultra-high-density plates

**Figure 1:**
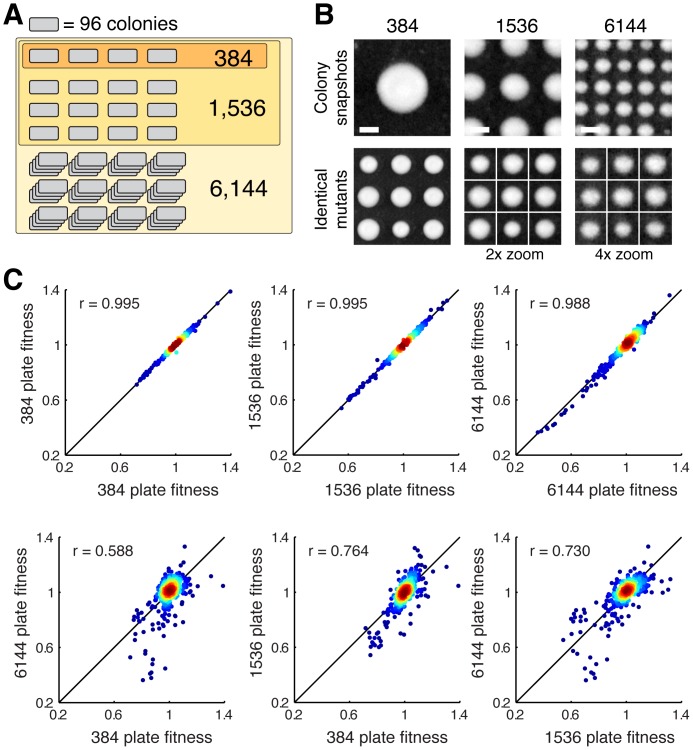
Experimental design and correlations between different colony densities. (**A**) Diagram indicating the overlap in yeast strains used in the various-colony formats – mutants in lower density plates are always included in higher-density plates. (**B**) Snapshots of 384-, 1536-, and 6144-colony plates (top row, scale bar 1 mm) and mosaic view of identical mutant colonies assembled from 1536- or 6144-colony plates (zoomed) and compared to the 384-colony plate growth (bottom row). (**C**) To compare data quality between formats, we correlated replicates of the different formats internally or across (same format median of N = 9 replicates each; across format median of N = 18 replicates each).

Current transfer pads only exist up to a maximum density of 1536 pins per pad. To increase colony density further, we developed a novel transfer pad with 6144 individual pinheads, allowing us to print plates with 6144 or 24576 clearly defined yeast colonies (**Material and Methods**). To compare the various density performances, we grew single gene deletion mutants from the haploid yeast knockout strain collection in 384-, 1536-, and 6144-colony grids in large plate replicate numbers for 48 hrs (N = 18). Mutant colonies grown at lower densities were a subset of those grown at higher densities, allowing us to compare the same 384 yeast mutant fitness values across densities (**Fig. 1**). Colony sizes were measured and analyzed as described in the **Material and Methods**.

**Figure 2:**
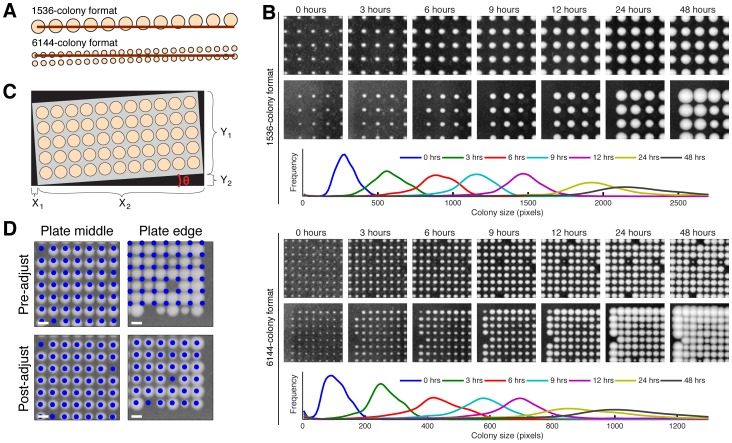
Colony growth kinetics and colony grid alignment. (**A**) Diagram of rows of larger and smaller colonies, each angled at 0.5 degrees relative to the reference (horizontal bars). Small errors in image rotation in the 6144-colony plates can lead to substantial colony identification errors. (**B**) Time-lapse imaging of the current 1536-density (above) and the new super-high-density format (below) reveals optimal imaging time points of 24–48 hrs for the 1536 and 12–24 hrs for the 6144 format (identical scale for all images). (**C**) Geometric solution for the image rotation problem. Given that the corners of the plate touch the edges of the cropped image, the width and height of the image can each be decomposed into the sum of two smaller values. These four values (X_1_, X_2_, Y_1_, Y_2_) are all trigonometric functions of *θ*, the angle of orientation of the grid, and the width and height of the plate. These functional relationships comprise a non-linear system of equations with a closed-form solution, which we solved for *θ.* (**D**) Snapshots of colonies growing in the middle and on the edge of 6144-colony plates; blue dots indicate the positions of the grid before (upper row) and after (lower row) the grid-adjustment step (scale bars 1 mm).

Growing 6144 yeast colonies on a single agar plate introduces unique challenges in plate image analysis, rendering previous data extraction approaches inadequate [6], [10]–[13], and led us to develop a new image analysis and data normalization software package (available at https://github.com/brazilbean/Matlab-Colony-Analyzer-Toolkit). Small errors in orientation in the 6144 format are sufficient to misalign colonies at the ends of each row or column due to the increased proximity of colonies (**Fig. 2A**). Consequently, we needed an algorithm that could precisely identify the locations of each colony in the 6144-grid, which is particularly challenging with small colonies (i.e. 0- or 3-hrs time points, **Fig. 2B**). We achieved a highly-accurate grid alignment by estimating the angle of grid orientation using the aspect ratio of the cropped image (**Fig. 2C** and **Note S1**), estimating the locations of the four corners of the grid based on grid dimensions and spacing, and iteratively interpolating the remaining locations of the grid/colony positions by describing each location as a linear function of the grid row and column positions [6], [13] (**Fig. 2D**).

**Figure 3:**
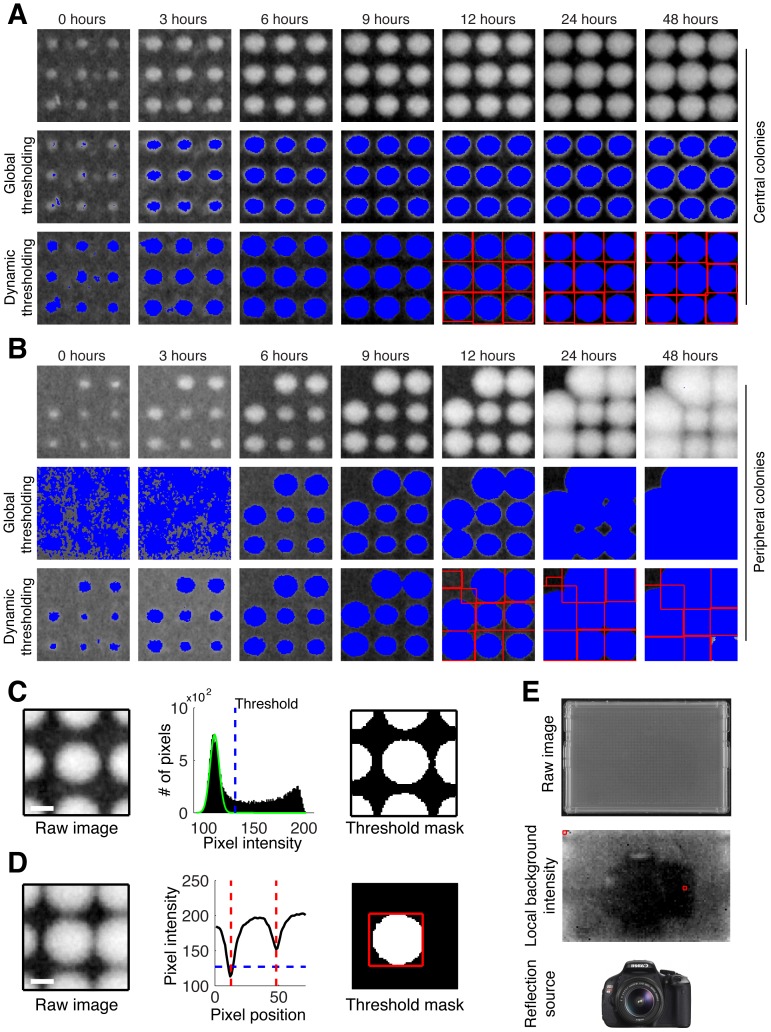
Comparison of global and dynamic intensity threshold algorithms. (**A, B**) Snapshots of colonies in the plate center and periphery, respectively, 0, 3, 6, 9, 12, 24, and 48 hours; blue pixels in the middle and bottom rows of each panel indicate the pixels called by the respective algorithm as foreground (i.e. colony as opposed to background); red lines indicate predicted colony boundaries. The global intensity used in (A) was computed on the peripheral window, while the global intensity used in (B) was computed on the central window, highlighting the problems of global tresholding. (**C**) Gray-scale snapshots of a single colony at 12 hours (left, scale bar 500 µm); histograms showing the distribution of pixel intensities for the snapshot, the green curve represents the normal distribution fit to the leftmost peak (indicating the distribution of background pixel intensities), blue dotted lines indicate the threshold used to distinguish colony from background (middle); binary output (right, threshold applied) (**D**) Gray scale snapshot centered on an overgrown colony (left, scale bar 500 µm); line plot of median pixel intensity across the center of the snapshot (middle, blue line indicates local intensity threshold, red line indicates the colony boundary); binary image with intensity threshold and bounding box applied (right). (**E**) Local dynamic background estimation is very sensitive and allows for accurate colony-size estimations across a large background intensity range. Original photo of a 0-hour 6144 plate (top); grey scale heat map of the estimated background intensity for each grid position in the 0-hour image (middle; red boxes indicate the positions of the central and peripheral snapshots shown in [A,B]); the reflection of the camera used (bottom) is clearly captured by the background intensity estimation algorithm, demonstrating its sensitivity.

Previous algorithms [6] relied on a single plate-wide pixel-intensity threshold to measure colony sizes, an approach we found to be inaccurate leading to over- and under-estimation of small colony sizes. This effect was particularly obvious at early time points with small colonies (0–3 hrs ‘Global thresholding’ **Fig. 3A,B**) and at late time points for peripheral colonies (24–48 hrs ‘Global thresholding’ **Fig. 3B**). We overcame this problem by implementing an algorithm that determines a dynamic pixel-intensity cutoff for each colony based on the distribution of local background pixel-intensities (**Fig. 3C**). Additionally, when colonies are overgrown, the pixels between adjacent colonies may have intensities greater than the threshold, resulting in multiple colonies being counted as one, a problem we solved by determining the local minima surrounding each colony (**Fig. 3D**). In summary, we developed an image-processing pipeline with an improved ability to correctly identify colonies over a large dynamic range of colony sizes, using sensitive local background detection (**Fig. 3E** **and** **4**) and improved colony identification algorithms.

**Figure 4:**
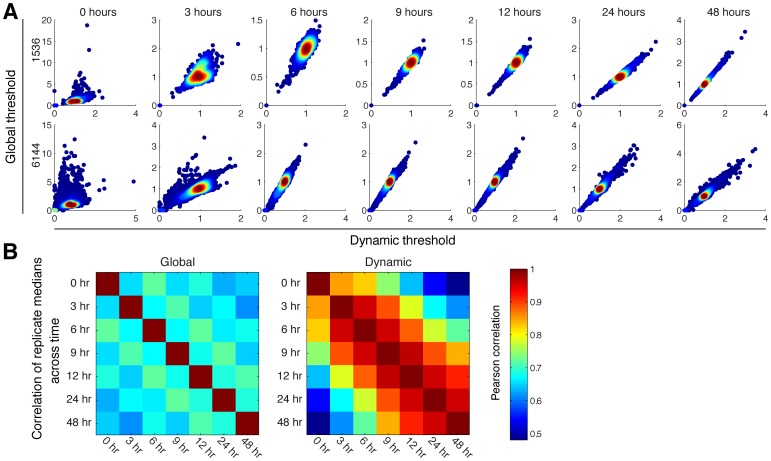
Effect of global *versus* dynamic background. (**A**) Comparison of globally or locally/dynamically tresholded colony sizes. While good correlation is achievable between 6 and 24 hrs, poor correlation is observed at the extreme ends of the experiment. (**B**) In general, using dynamic local thresholding (right) achieves much better data correlation across time-points than global thresholding (left).

### Growth performance, cost, and signal quality of ultra-high-density plates

**Figure 5:**
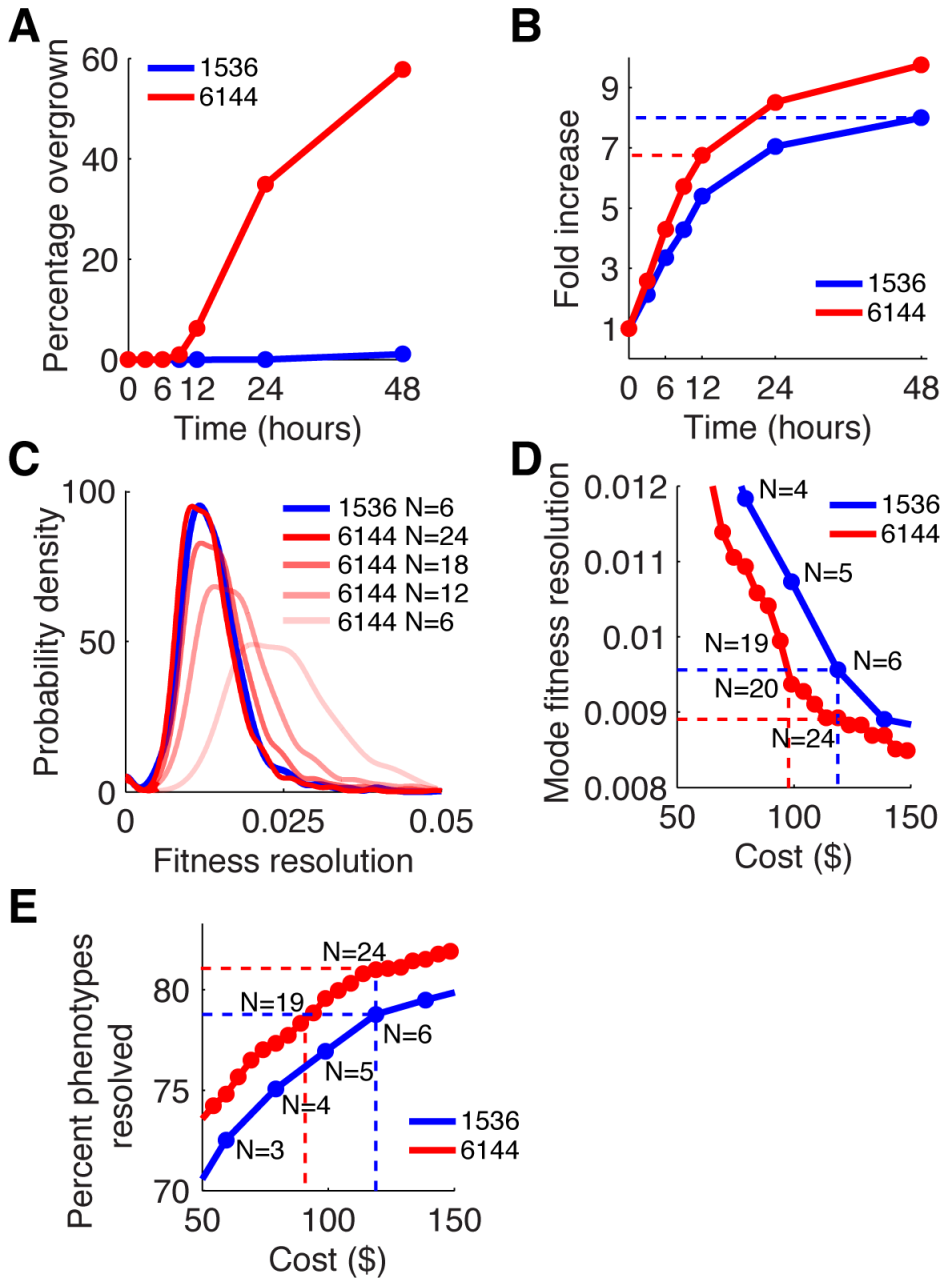
Ultra-high-density format data quality and cost efficiency. (**A**) Percentage of colonies overgrown. (**B**) Growth curves based on median colony area fold-increase since pinning (dashed lines indicate fold increase at 12 hrs [6144] or 48 hrs [1536], N = 18 for each colony density). (**C**) Distribution of fitness resolutions for 1536 and 6144 format (N =  replicate numbers). (**D**) Mode fitness resolutions for a given cost/replicate level (dashed lines indicate equal cost/quality levels, N =  replicate numbers). (**E**) Percentage of single mutants that can be resolved (dashed lines indicate equal cost/quality levels, N =  replicate numbers).

Utilizing our image analysis software package, we then compared the growth and noise performance of 1536-colony and 6144-colony formats (referred to as *1536* and *6144* formats). While typical screens in the 1536 format are imaged 48 hours after pinning, we found that plates in the 6144 format begin to overgrow after 12 hours (**Fig. 5A**); therefore, we make all our comparisons between the 1536/48-hour images and the 6144/12-hour images. Despite the decreased incubation time, 6144 plates exhibit a comparable fold-growth to 1536 plates (∼7-fold versus ∼8-fold, **Fig. 5B**), because the much smaller pin heads on the 6144 pads produce smaller starting colony sizes (**Fig. S1A–C**).

To benchmark the usability of our new technology, we performed colony-based fitness measurements on yeast gene-deletion mutants, identical to the technique used in epistasis or chemo-genetic screens. The goal of any fitness-based genetic screen is to establish which mutants exhibit a significant deviation from expected fitness levels. The fitness variance across replicates is a crucial parameter that determines what minimal fitness difference (“fitness resolution”) can be called significant. We observed that the variance of colony measurements increased as plate density increased, and decreased as colonies were grown for a longer time (**Fig. S1D**). Consequently, the 6144 format suffers from lower fitness resolution than the 1536 format, assuming identical numbers of replicates (1536 and 6144 N = 6, **Fig. 5C**). However, the 6144 format's higher density allows more replicates to be run at equal or lower cost, which can increase 6144 fitness resolution to be as good or even better than the 1536 fitness resolution (6144 N = 12, 18, 24, **Fig. 5C**).

Additionally, the dynamic range of fitness levels was slightly larger in 6144 compared to 1536 plates (**Fig. S1E**), indicating that 6144 plates could be cost and quality competitive. To explore the relationship between cost (i.e. replicate number) and fitness resolution, we calculated the minimum fitness phenotype resolvable as a function of the number of replicates and found 6144 plates to be less expensive at equal quality (17% cost reduction). Conversely, better quality could be achieved at equal cost (+7% fitness resolution) compared to 6 replicates of 1536 plates (**Fig. 5D**). We also calculated the fraction of single mutants found significantly different from wildtype at a specific replicate number or cost level (p<0.05, **Fig. 5E**) and observed that the 6144 format can be used to increase the percentage of mutants with a significant phenotype at equal cost (+2.5%), or to allow for cost savings at equal identification level (25% cost reduction, **Fig. 5E**). Importantly, 6144 plates allow the experimenter to dramatically decrease expenses without risking statistical errors that arise with small numbers of replicates. For example, a typical experiment might run 6 replicates in the 1536 format, or 18 replicates in the 6144 format; however, reducing the number of replicates by a factor of 3, 2 replicates in the 1536 format would produce data subject to statistical errors, while 6 replicates of the 6144 plates would not, causing only a mild degradation in data quality. In summary, 6144 plates can be used to improve data quality at equal cost, to cut cost by about 15–20% while maintaining equal data quality, or to finely choose the quality-for-cost balance for a given experiment, while substantially reducing screening time in all cases (−75%, 12 hrs vs. 48 hrs).

### Ultra-high-density plates from high-density pads

**Figure 6:**
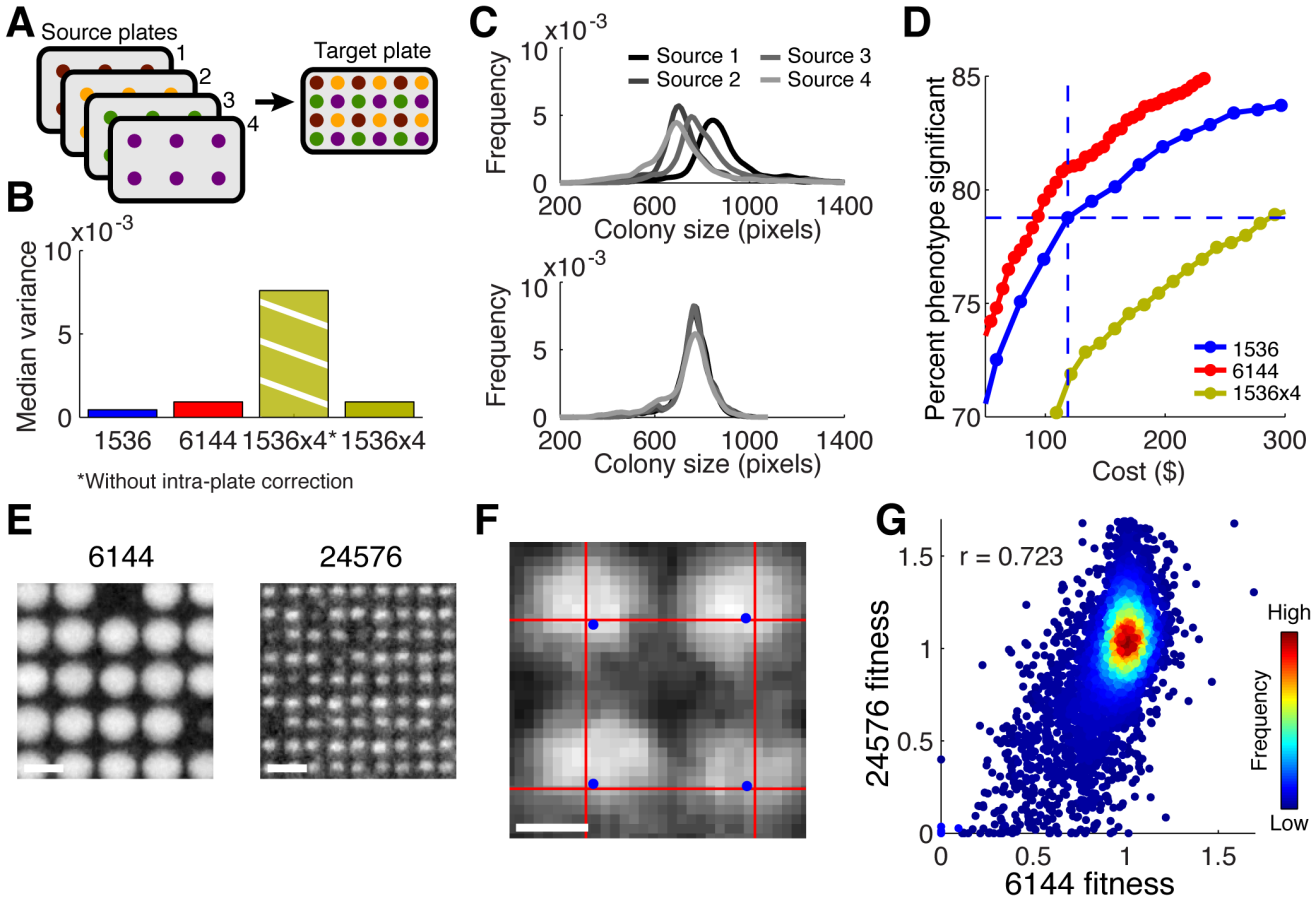
Up-scaling and hyper-density. (**A**) Schematic of the effects of up-scaling: the combination of different lower-density source plates into one higher-density target plate. (**B**) Comparison of variance in plates pinned with dedicated density-pads (1536, 6144) and plates using up-scaling with or without intra-plate source correction. (**C**) Colony size distributions obtained by the analysis pipeline without (top) and with (bottom) the intra-plate source correction. (**D**) Comparison of the percentage of single mutants that can be identified with a significant fitness phenotype at a given cost/replicate level (--- indicate N = 6 at 1536 density). (**E**) Snapshots of 6144- and 24,576 hyper-density colonies at equal scale (scale bar 1 mm). (**F**) Zoomed image showing jitter effect on colony placement; red grid represents perfect alignment, blue dots denote actual pin position (scale bar 100 µm). (**G**) Correlation of fitness measurements obtained with ultra-high- and hyper-density plates.

Higher-density plates can be produced from lower density source plates by pinning multiple source plates onto a common target plate, a process we call “up-scaling” (**Fig. 6A**). We examined whether it is possible to use 1536 pads to create 6144 plates with the same beneficial properties as described above. We found that 6144 plates pinned with 1536 pads (“1536x4”) and analyzed *via* the normal data analysis pipeline showed a dramatic increase in variance (**Fig. 6B**, “1536x4 without intra-plate correction”). Further analysis revealed that most of this variance increase was due to batch effects unique to each source plate that were preserved in the target plate (**Fig. 6C**, top). Analyzing the colonies pertaining to each source plate as separate, lower-density plates improved the variance dramatically (**Fig. 6C**, bottom, and **Fig. 6B**, “1536x4”). Overall, up-scaling using 1536-pads yields growth-curves, dynamic ranges, and quality levels close to 6144 pad pinned plates (**Fig. S2**). However, four pinning pads are used to up-scale for each ultra-high-density plate, thus cost benefits cannot be realized using up-scaling (**Fig. 6D**, assuming a cost ratio of 2∶1 for plates to pads and equal cost per pad).

### Feasibility of hyper-density plates with 24576 individual colonies

Here we have described the technical qualities of the emerging 6144 format we developed for yeast high-throughput screening, and one could certainly consider the possibility of future increases in colony-density. Given the promising findings on data quality using up-scaling, we explored the technical feasibility of pinning 24576 yeast colonies onto a single agar plate using 6144 pads (“hyper-density plates”). Using a modified 6144 pinning protocol, we successfully created plates with this tremendous colony density, allowing us to run a genome-wide single mutant screen with four replicates on a single agar plate (**Fig. 6E**). At such small scales the spatial precision required to place pinheads in perfect geometric alignment (to avoid uneven colony neighbor distances) increases. However, we found colony alignment to err by less than one pixel on average (**Fig. 6F**). The resulting fitness values, while increasingly noisy, nevertheless correlate well with lower density data (**Fig. 6G**). At these densities, colonies are only 100–200 µm in diameter and new technical challenges arise: colony images become pixelated, resulting in more noise, and the contrast between colony and background pixel intensity decreases, making colony detection more difficult. Furthermore, colonies arrayed at this density begin to overgrow within 3 to 6 hours, reducing the amount of time for growth-defects to be manifest. However, we believe that this hyper-density format could find useful screening applications, especially when combined with further improvements in data acquisition and analysis.

## Discussion

Using a heavily utilized genetic screening system for benchmarking, our analysis suggests that the new 6144 format can be used to effectively cut experimental costs and duration while still maintaining the same level of discriminatory power and quality as the old 1536 format. The new format also gives the researcher more flexibility to choose the desired cost-quality balance with dramatic cost savings possible (>50%). Our study of the feasibility of hyper-density plates with 24576 individual yeast colonies shows that simultaneous fourfold-coverage of the whole yeast genome on a single agar plate at reasonable quality-levels is now technologically possible.

Our study suggests that our ultra-high-density pad and analysis pipeline exceeds the technical standards necessary to produce high quality data in different types of screens that are based on the quantification of individual microbial colonies. While recent developments in genetic screening technology have enabled researchers to perform some genetic screening experiments in pooled, liquid form [14], [15] many “omics” screening technologies have or cannot be adapted to a this type of format, and these screens will undoubtedly benefit from the availability of the ultra-high-density pads.

Intriguingly, our results suggest that if extreme data quality were desired and cost irrelevant, one could pin low-density plates using higher-density pads (e.g. only every 4^th^ or every 16^th^ colony pinned), allowing for the benefits of very large fold-increases of growth (due to small pin heads) to be combined with longer incubation times leading to colony sizes with small biological and technical noise. These considerations and other experimental techniques may be useful for further extending the limits of high-throughput screening technology.

Data quality could also be improved through modifications to the image acquisition system. As the purpose of this study was to compare 1536- and 6144-format experiments, we did not conduct thorough investigations into the relative merits of image acquisition improvements. However, adjustments in the number of megapixels, lens types, and aperture settings could be made to accomplish incremental improvements in image and data quality. Similarly, researchers desiring to use the 6144-format pads should still be able to acquire data of reasonable quality even if some element of their imaging system is not the same as ours (e.g. their camera provides 10Mpixels instead of 18Mpixels).

### Glossary

**epistasis** – in double-mutant genetic screens, epistasis occurs when the phenotype of the double mutant deviates from the predicted combination of the single mutant phenotypes; epistasis is indicative of functional relationships among the genes perturbed by the mutations.

**fitness** – in genetic screens, fitness refers to the ratio of the mutant phenotype (i.e. quantitative measurement, typically growth rate) to the control phenotype; e.g. a mutant with a fitness of 0.9 grows at 90% the growth rate of the control strain.

**fitness difference** – the difference in fitness between strains

**fitness variance** – the variance of repeated measurements of a strain's fitness

**fitness resolution** – the minimum fitness difference needed to determine with statistical confidence that two strains exhibit different fitness phenotypes; greater dynamic range among possible fitness values and smaller fitness variance improve fitness resolution.

**hyper-density** – 24576 colony format

**ultra-high density** – 6144 colony format

## Supporting Information

Figure S1 **Colony size, overgrowth, variance, and dynamic range over time.** (**A**) Comparison of the different pin pad formats (6144, 1536, 384, 96; left to right). (**B**) Median colony size over time. (**C**) Percentage of plate overgrown over time. (**D**) Standard deviation between replicates over time. (**E**) Phenotype (fitness) dynamic range over time.
(JPG)

Figure S2 **Colony size, overgrowth, variance, and dynamic range over time including 1536x4** (**A**) Median colony size over time. (**B**) Percentage of plate overgrown over time. (**C**) Standard deviation between replicates over time. (**D**) Phenotype (fitness) dynamic range over time.
(JPG)

Note S1 **Derivation of** ***θ*** **.**
(PDF)
