# Supplementary figures and images for: Development of Ultra-High-Density Screening Tools for Microbial “Omics”
Source: PLoS One. 2014 Jan 21;9(1):e85177. doi: 10.1371/journal.pone.0085177 (PMC3897414; doi:10.1371/journal.pone.0085177)

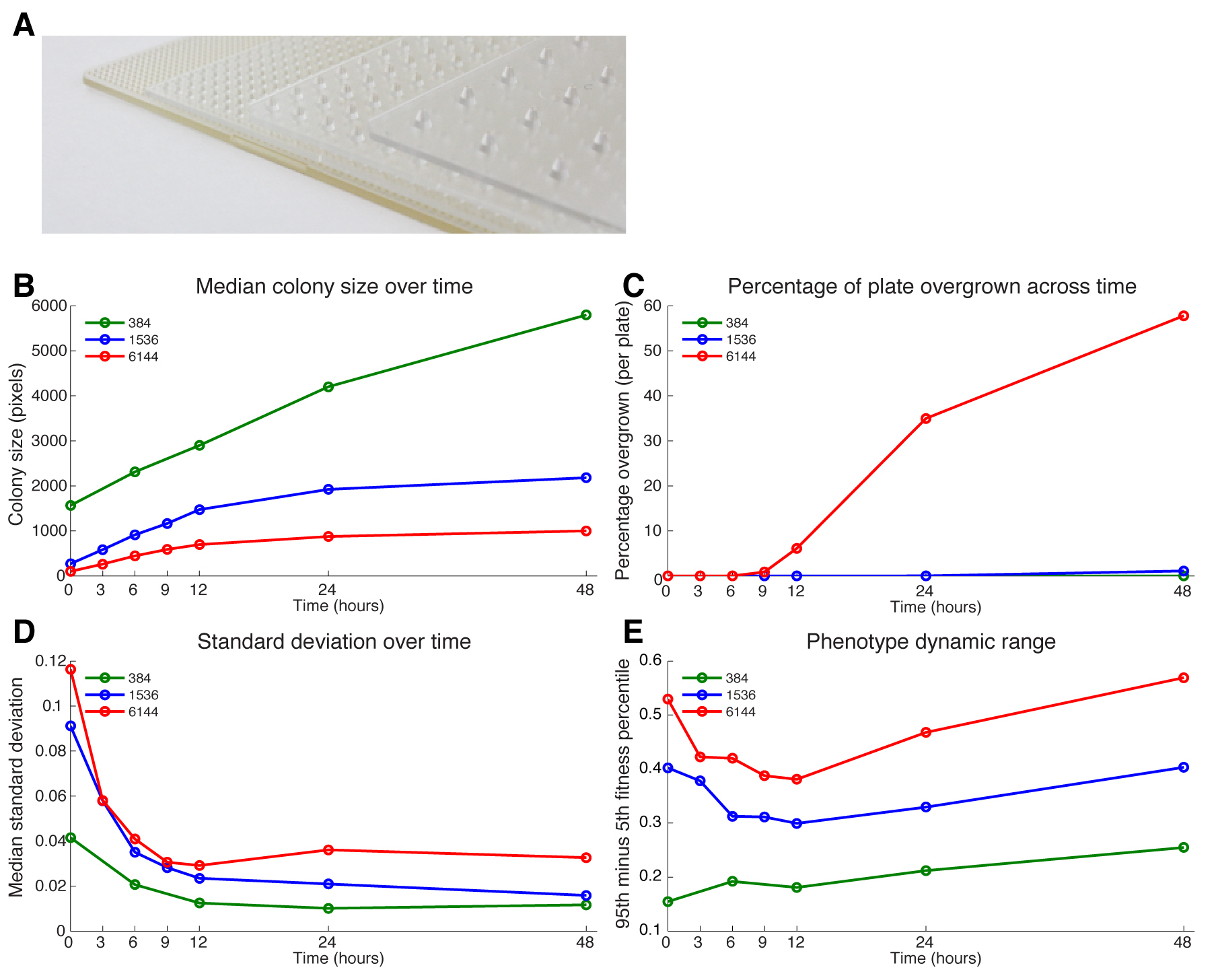

Supplement: Figure S1 — Colony size, overgrowth, variance, and dynamic range over time. (A) Comparison of the different pin pad formats (6144, 1536, 384, 96; left to right). (B) Median colony size over time. (C) Percentage of plate overgrown over time. (D) Standard deviation between replicates over time. (E) Phenotype (fitness) dynamic range over time. (JPG) [file pone.0085177.s001.jpg]

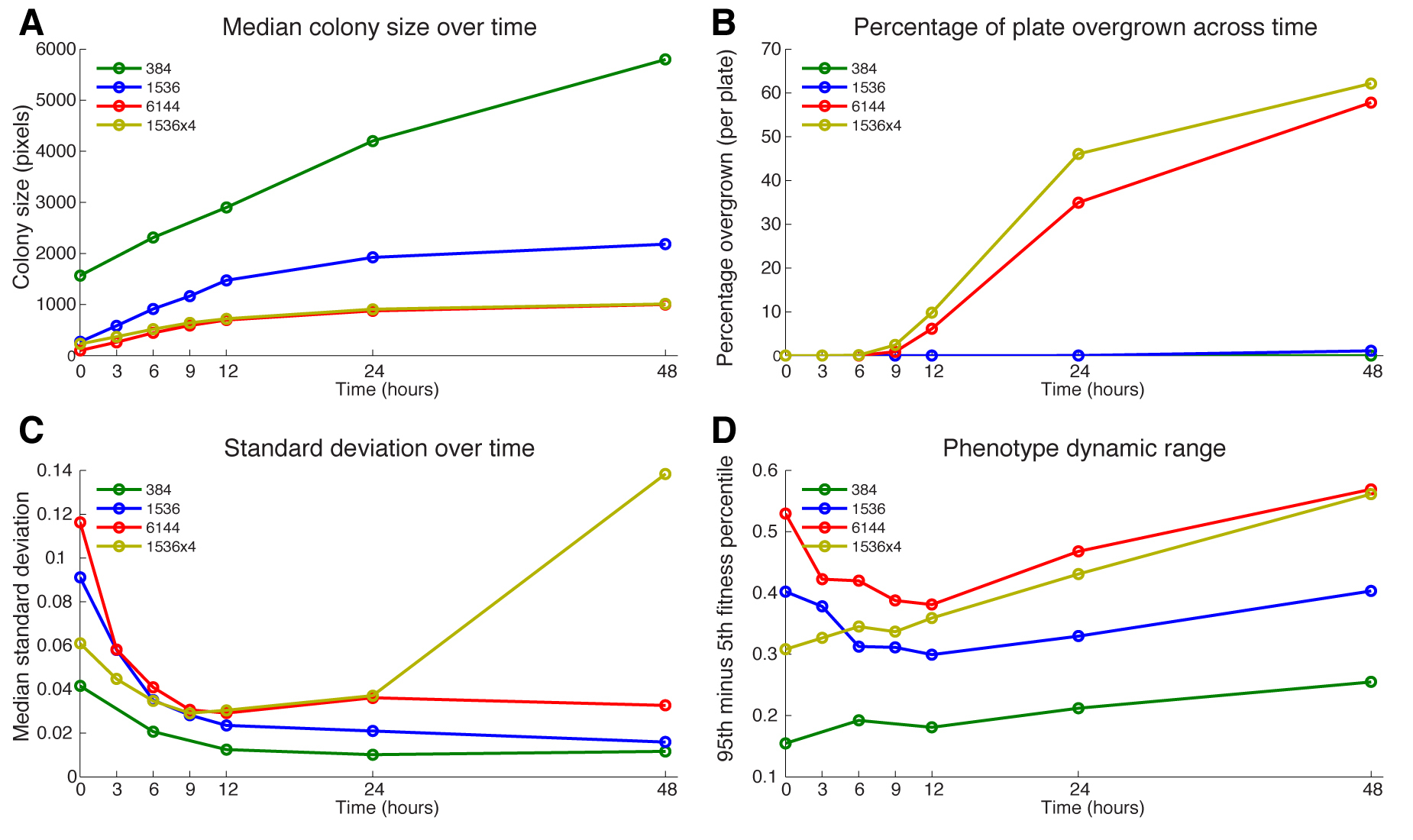

Supplement: Figure S2 — Colony size, overgrowth, variance, and dynamic range over time including 1536x4 (A) Median colony size over time. (B) Percentage of plate overgrown over time. (C) Standard deviation between replicates over time. (D) Phenotype (fitness) dynamic range over time. (JPG) [file pone.0085177.s002.jpg]
