# Supplementary material for: Development of Ultra-High-Density Screening Tools for Microbial “Omics”
Source: PLoS One. 2014 Jan 21;9(1):e85177. doi: 10.1371/journal.pone.0085177 (PMC3897414; doi:10.1371/journal.pone.0085177)
Supplement: Note S1 — Derivation of θ . (PDF) [file pone.0085177.s003.pdf]

Supplemental Note 1: Derivation of θ

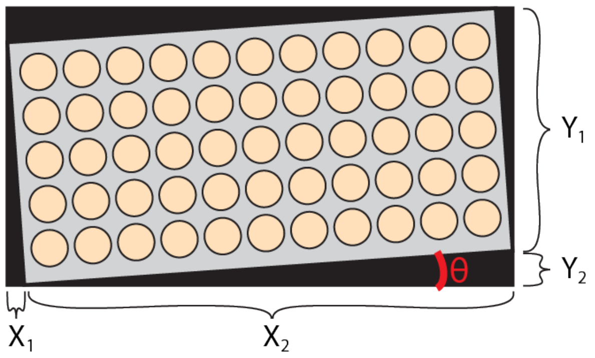

$$x = x_1 + x_2$$
$$y = y_1 + y_2$$

$$x_1 = h \cdot \sin\theta$$
$$x_2 = w \cdot \cos\theta$$
$$y_1 = h \cdot \cos\theta$$
$$y_2 = w \cdot \sin\theta$$

$$x = h \cdot \sin\theta + w \cdot \cos\theta$$
$$y = h \cdot \cos\theta + w \cdot \sin\theta$$

$$\frac{x}{\cos\theta} = h \cdot \tan\theta + w$$
$$\frac{y}{\cos\theta} = h + w \cdot \tan\theta$$

$$\frac{x}{h \cdot \tan\theta + w} = \cos\theta = \frac{y}{h + w \cdot \tan\theta}$$

$$x \cdot (h + w \cdot \tan\theta) = y \cdot (h \cdot \tan\theta + w)$$

$$x \cdot h + x \cdot w \cdot \tan\theta = y \cdot h \cdot \tan\theta + y \cdot w$$

$$\tan\theta \cdot (x \cdot w - y \cdot h) = y \cdot w - x \cdot h$$

$$\tan\theta = \frac{y \cdot w - x \cdot h}{x \cdot w - y \cdot h}$$

$$\tan\theta = \frac{y - x \cdot \frac{h}{w}}{x - y \cdot \frac{h}{w}}$$

Given that the corners of the plate touch the edges of the cropped image, the width and height of the image can each be decomposed into the sum of two smaller values. These four values are all trigonometric functions of θ, the angle of orientation of the grid, and the width and height of the plate. These functional relationships comprise a non-linear system of equations with a closed-form solution, which we solved for θ:

X and Y (the width and height of the image, in pixels) are known. The ratio of the height to the width of the plate, $\frac{h}{w}$, is also known.

The height, h, and width, w, of the plate, in pixels, change depending on the resolution of the image (which depends on the megapixel capacity and positioning of the camera). However, the ratio of h to w is constant for all image resolutions – this is why the final form uses the ratio of h to w.
